# Supplementary material for: School absenteeism among children and adolescents aged 6–19 years with sickle cell disease in Uganda: A comparative cross-sectional study
Source: PLoS One. 2026 May 29;21(5):e0350308. doi: 10.1371/journal.pone.0350308 (PMC13221043; doi:10.1371/journal.pone.0350308)
Supplement: S1 Table — This is the S1 Fig legend. (DOCX) [file pone.0350308.s001.docx]

**Table S1. Socio-demographics for caregivers of children with Sickle cell disease included in the study**

| **Characteristic** | **n (%) or Median (IQR)** |
| --- | --- |
| **Sex**  Female  Male | 158 (88.3)  21 (11.7) |
| **Age**  Median (IQR), years | 38 (32-45) |
| **Relationship with child with SCD**  Biological parent  Step mother/father  Uncle/ aunt  Sibling  Cousin  Grandmother | 142 (79.3)  2 (1.1)  11 (6.1)  9 (5.0)  2 (1.1)  13 (7.3) |
| **Marital status**  Single  Co-habiting  Married  Separated  Widow/ widower | 30 (16.8)  40 (22.4)  73 (40.8)  21 (11.7)  15 (8.4) |
| **Biological parents’ survival status**  Both alive  Only father alive  Only mother alive  Both not alive | 157 (87.7)  5 (2.8)  15 (8.4)  2 (1.1) |
| **Parents co-habiting**  No  Yes | 91 (50.8)  88 (49.2) |
| **Number of children in the home**  Median (IQR) | 4 (3-6) |
| **Number of children with SCD**  Median (IQR) | 1 (1-2) |
| **Total household income (USD)**  Median (IQR) | 81 (54-162) |
| **Income sufficient to pay school fees**  No  Yes | 132 (73.7)  47 (26.3) |
| **Children missing school** **due to fees**  No  Yes | 50 (27.3)  129 (72.1) |
