# Supplementary material for: School absenteeism among children and adolescents aged 6–19 years with sickle cell disease in Uganda: A comparative cross-sectional study
Source: PLoS One. 2026 May 29;21(5):e0350308. doi: 10.1371/journal.pone.0350308 (PMC13221043; doi:10.1371/journal.pone.0350308)
Supplement: S2 Table — This is the S2 Fig legend. (DOCX) [file pone.0350308.s002.docx]

**Table S2. Socio-economic status of the biological parents of the children with Sickle cell disease included in the study**

| **Characteristic** | **Father (N=149)**  **n (%) or Median (IQR)** | **Mother (N=169)**  **n (%) or Median (IQR)** |
| --- | --- | --- |
| **Education level**  None  Nursery  Primary  Secondary  Tertiary | 1 (0.7)  0 (0.0)  38 (25.5)  78 (52.4)  32 (21.5) | 4 (2.4)  1 (0.6)  51 (30.2)  84 (49.7)  29 (17.2) |
| **Current occupation status**  Unemployed  Self employed  Professional  Other | 12 (8.1)  60 (40.3)  35 (23.5)  42 (28.2) | 39 (23.1)  77 (45.6)  22 (13.0)  31 (18.3) |
| **Monthly income (USD)**  Median (IQR) | 135* (81-270) | 64.8** (36.5-108) |
| **Type of job**  None  Full time job  Part time job  Casual laborer  Other | 14 (9.4)  116 (77.9)  9 (6.0)  8 (5.4)  2 (1.3) | 40 (23.7)  102 (60.4)  16 (9.5)  10 (5.9)  1 (0.6) |

Note. *n=75, **n=108
