# Supplementary material for: School absenteeism among children and adolescents aged 6–19 years with sickle cell disease in Uganda: A comparative cross-sectional study
Source: PLoS One. 2026 May 29;21(5):e0350308. doi: 10.1371/journal.pone.0350308 (PMC13221043; doi:10.1371/journal.pone.0350308)
Supplement: Attachment — Submitted filename: Response to Reviewers.docx [file pone.0350308.s003.docx]

**MAKERERE** **UNIVERSITY**

**COLLEGE OF HEALTH SCIENCES**

**SCHOOL OF MEDICINE**

**DEPARTMENT OF PEDIATRICS AND CHILD HEALTH**

24^th^ February 2026

Dr. Aloysius Gonzaga Mubuuke
Academic Editor
PLOS One

Dear Dr. Mubuuke,

On behalf of my co-authors, I hereby submit the responses to the reviewers’ comments for the manuscript titled **“School absenteeism among children and adolescents aged 6–19 years with sickle cell disease in Uganda: a comparative cross-sectional study”** (Manuscript ID: **PONE-D-25-63605**). We thank the reviewers for the careful review of our manuscript and for the constructive guidance provided. We have addressed each comment in detail below and revised the manuscript accordingly.

**JOURNAL REQUIREMENTS**

**Journal Requirement 1: PLOS ONE Style and File Naming**

***Comment:*** ***1. When submitting your revision, we need you to address these additional requirements.***

***Please ensure that your manuscript meets PLOS ONE's style requirements, including those for file naming. The PLOS ONE style templates can be found at***

[***https://journals.plos.org/plosone/s/file?id=wjVg/PLOSOne_formatting_sample_main_body.pdf***](https://journals.plos.org/plosone/s/file?id=wjVg/PLOSOne_formatting_sample_main_body.pdf) ***and***

[***https://journals.plos.org/plosone/s/file?id=ba62/PLOSOne_formatting_sample_title_authors_affiliations.pdf***](https://journals.plos.org/plosone/s/file?id=ba62/PLOSOne_formatting_sample_title_authors_affiliations.pdf)

Response: We confirm that the manuscript has been revised to fully comply with PLOS ONE formatting and style requirements, using the official PLOS ONE templates for the main body and title page. File naming conventions have also been updated in accordance with journal guidelines. All submitted files, including the revised manuscript with tracked changes, the clean manuscript, and its supporting information. In addition, we have adhered to the required naming and formatting standards.

**Journal Requirement 2: Funding Statement - Completeness**

***Comment 2. Thank you for stating in your Funding Statement:***

***This study was supported by the National Heart, Lung, and Blood Institute of the National Institutes of Health under Award Number D43TW012466. The content is solely the responsibility of the authors and does not necessarily represent the official views of the National Institutes of Health.***

***Please provide an amended statement that declares *all* the funding or sources of support (whether external or internal to your organization) received during this study, as detailed online in our guide for authors at http://journals.plos.org/plosone/s/submit-now. Please also include the statement “There was no additional external funding received for this study.” in your updated Funding Statement.***

***Please include your amended Funding Statement within your cover letter. We will change the online submission form on your behalf.***

Response: The Funding Statement has been amended to declare all sources of support and now explicitly includes the statement indicating that no additional external funding was received.

Revised text: Cover letter

“This study was supported by the National Heart, Lung, and Blood Institute of the National Institutes of Health under Award Number D43TW012466. The content is solely the responsibility of the authors and does not necessarily represent the official views of the National Institutes of Health. There was no additional external funding received for this study.”

**Journal Requirement 3: Role of the Funder**

***Comment 3. Thank you for stating the following financial disclosure:***

***This study was supported by the National Heart, Lung, and Blood Institute of the National Institutes of Health under Award Number D43TW012466. The content is solely the responsibility of the authors and does not necessarily represent the official views of the National Institutes of Health.***

***Please state what role the funders took in the study. If the funders had no role, please state: "The funders had no role in study design, data collection and analysis, decision to publish, or preparation of the manuscript."***

***If this statement is not correct you must amend it as needed.***

***Please include this amended Role of Funder statement in your cover letter; we will change the online submission form on your behalf.***

Response: We confirm that the funders had no role in the conduct of the study. The Role of the Funder statement has been clarified accordingly.

Revised text: Cover letter

“The funders had no role in study design, data collection and analysis, decision to publish, or preparation of the manuscript.”

**Journal Requirement 4: Funding Information in Acknowledgments**

***Comment 4. Thank you for stating the following in the Acknowledgments Section of your manuscript:***

***I thank the research participants and clinical staff at the Sickle Cell Clinic at Mulago Hospital. I am grateful to the research assistants for collecting the data. Special thanks to Mirembe Vanessa and Kataike Peace for their commitment and hard work. I also appreciate the ENRICH program for the guidance and financial resources throughout this journey.***

***We note that you have provided funding information that is not currently declared in your Funding Statement. However, funding information should not appear in the Acknowledgments section or other areas of your manuscript. We will only publish funding information present in the Funding Statement section of the online submission form.***

***Please remove any funding-related text from the manuscript and let us know how you would like to update your Funding Statement. Currently, your Funding Statement reads as follows:***

***This study was supported by the National Heart, Lung, and Blood Institute of the National Institutes of Health under Award Number D43TW012466. The content is solely the responsibility of the authors and does not necessarily represent the official views of the National Institutes of Health.***

***Please include your amended statements within your cover letter; we will change the online submission form on your behalf.***

Response: We agree and have removed all funding-related text from the Acknowledgments section. The Acknowledgments now include only expressions of gratitude to participants, clinical staff, and research assistants. All funding information is presented exclusively in the Funding Statement, as per PLOS ONE policy.

Revised text: Section: Acknowledgements, pages-21, lines 402-404

“We thank the research participants and clinical staff at Mulago Hospital’s Sickle cell clinic. In addition, we are grateful to the research assistants for collecting the data. Special thanks to Mirembe Vanessa and Kataike Peace for their commitment and hard work.”

**Journal Requirement 5: Data Availability and Repository**

**Comment 5. Thank you for uploading your study's underlying data set. Unfortunately, the repository you have noted in your Data Availability statement does not qualify as an acceptable data repository according to PLOS's standards.**

**At this time, please upload the minimal data set necessary to replicate your study's findings to a stable, public repository (such as figshare or Dryad) and provide us with the relevant URLs, DOIs, or accession numbers that may be used to access these data. For a list of recommended repositories and additional information on PLOS standards for data deposition, please see https://journals.plos.org/plosone/s/recommended-repositories.**

Response: We thank the journal for this guidance. The minimal dataset required to replicate the study findings has now been deposited in a stable, public repository compliant with PLOS ONE standards, Figshare. The Data Availability Statement has been updated to include the correct repository name and DOI/URL.

Revised text: Availability of data and materials and references, pages: 20, lines-383-384

‘’The data described in this article can be freely and openly accessed at the Figshare repository (Nattimba, Juliane Peninah (2026). School absenteeism and associated factors among children and adolescents attending sickle cell clinic, Mulago National Referral hospital, Kampala district.. figshare. Dataset. <https://doi.org/10.6084/m9.figshare.31290772.v1>)’’

**Journal Requirement 6: Ethics Statement Location**

***Comment 6. Your ethics statement should only appear in the Methods section of your manuscript. If your ethics statement is written in any section besides the Methods, please delete it from any other section***

Response: We confirm that the ethics statement now appears only in the Methods section. Any duplicate ethics-related text appearing elsewhere in the manuscript has been removed.

**Journal Requirement 7: Supporting Information Files**

***Comment 7. We notice that your supplementary tables are included in the manuscript file. Please remove them and upload them with the file type 'Supporting Information'. Please ensure that each Supporting Information file has a legend listed in the manuscript after the references list.***

Response: All supplementary tables have been removed from the main manuscript and uploaded as separate Supporting Information files. Each Supporting Information file is now referenced appropriately in the manuscript, with legends listed after the references section.

**Journal Requirement 8: Reviewer-Recommended Citations**

***Comment 8. If the reviewer comments include a recommendation to cite specific previously published works, please review and evaluate these publications to determine whether they are relevant and should be cited. There is no requirement to cite these works unless the editor has indicated otherwise***

Response: We have now included an appropriate citation in the Methods section to support the statistical approach used for the sample size calculation. The reference has been added to clarify the methodological basis and improve the transparency and reproducibility of the study design. The data repository from which relevant comparative or contextual data were obtained has also been appropriately referenced. The reviewers recommended no additional citations.

Revised text: Methods- Data analysis- Page8, Lines 184-186, Availability of data and Materials-Page 20, Lines 390-391

“The assumed proportions (p₁ and p₂) were informed by prior studies reporting school absenteeism among children with SCD and comparison groups in similar contexts. (12)”

**EDITORIAL COMMENTS**

***Editor Comment 1:* *Please re-check the design which you call a cross-sectional design right from the title. You might have to reconsider giving it the appropriate name as a matched case-control study because this is what you did.***

Response: We respectfully maintain that the study is appropriately classified as a comparative cross-sectional study and not a case-control study. We recognize that the inclusion of two groups may resemble a case-control structure; however, the recruitment process and analytical framework align with cross-sectional methodology.

Participants were recruited based on exposure status (presence or absence of sickle cell disease) rather than outcome status (chronic absenteeism). Children with SCD were consecutively enrolled from the Sickle Cell Clinic during routine visits. For each enrolled child with SCD, a sibling or peer without SCD of similar age was recruited as a comparison participant. Importantly, participants were not selected based on whether they had chronic absenteeism, and absenteeism status was unknown at the time of enrollment.

Both exposure (SCD status) and outcome (chronic absenteeism during the previous school term) were assessed concurrently at a single point in time. No retrospective identification of cases and controls based on absenteeism occurred, and no matching or conditional analytical techniques typical of case-control designs were applied.

Revised text: Section**:** Methods - *Study design and setting*, Page**:**5, Lines:95-99

“This was a comparative cross-sectional study conducted among children aged 6–19 years. Participants were recruited based on sickle cell disease (SCD) status rather than absenteeism status. Children with SCD were consecutively enrolled during routine clinic visits at the national referral hospital’s Sickle Cell Clinic. For each enrolled child with SCD, a sibling or age-comparable peer without SCD was recruited from the same household or community. Chronic absenteeism was assessed at the time of the interview based on school attendance during the previous term. Participants were not selected based on absenteeism status, and no retrospective identification of cases and controls was performed.”

***Editor Comment 2: The authors should highlight key limitations of a case-control study which readers should be aware of.***

Response: We agree with the Editor that clearly articulating study limitations is essential. While this study is cross-sectional, we recognize that several limitations commonly discussed in observational and case-control studies are relevant. We have therefore expanded the Limitations subsection to address potential sources of bias, including recall bias, lack of temporality, and selection bias. In particular, we discuss the possibility of differential recall of school absenteeism among caregivers of children with sickle cell disease, as well as the exclusion of children who may have permanently withdrawn from school.

Revised text: Section: Discussion - *Limitations*, Pages**:** 18-19, Lines**:**344-362

“First, the cross-sectional design limits the ability to establish temporal relationships between associated factors and chronic absenteeism; therefore, causal inferences cannot be made. Second, school absenteeism was assessed using caregiver or participant self-report for the previous school term, which may be subject to recall bias and misclassification. Caregivers of children with sickle cell disease may recall illness-related absences more readily than caregivers of comparison participants, potentially resulting in differential recall. Third, as with many observational studies, residual confounding from unmeasured variables cannot be excluded despite multivariable adjustment. Factors such as school-level support systems, teacher perceptions, or household socioeconomic dynamics not fully captured in the study may influence absenteeism patterns. Finally, participants were recruited from a clinical setting and included only children currently enrolled in school. Children with sickle cell disease who had permanently withdrawn from school were not captured, which may have led to underestimation of the true extent of absenteeism among the children with SCD at the Mulago Hospital Sickle Cell clinic.”

***Editor Comment 3:* *The discussion, try to show how your work adds to the global discourse around sickle cell disease beyond the local study setting.***

Response: The Discussion has been substantially strengthened to situate our findings within the broader global literature on sickle cell disease and educational outcomes. We now compare our findings with studies from both high-income and low- and middle-income countries and highlight how structural constraints, such as limited access to remote learning and frequent healthcare utilization, may amplify educational disadvantage in resource-limited settings. We further emphasize that school absenteeism among children with sickle cell disease is a global concern, but one that manifests differently depending on health system capacity, educational infrastructure, and social support mechanisms. This framing enhances the relevance of our findings beyond Uganda.

Revised text: Section: Discussion, Page: 15–16, Lines:283-295

“The findings of this study contribute to the growing global discourse on the educational implications of sickle cell disease (SCD). Across both high-income countries and low- and middle-income countries, children living with SCD consistently experience higher levels of school absenteeism compared to their peers (1,2,22). However, the magnitude and consequences of absenteeism are shaped by contextual factors, including healthcare access, availability of school-based accommodations, and broader social support systems (2). In high-income settings, structured educational support services, individualized learning plans, and access to remote learning options may mitigate some of the academic disruption associated with recurrent illness and hospitalization (1). In contrast, in many resource-limited settings, including Uganda, limited access to compensatory academic support and fewer school-based health accommodations may amplify the afore reported cases of absenteeism among children with SCD in Africa. “

### *****Editor Comment 4:***** Lastly, the entire manuscript still needs proofreading to correct obvious language errors.

**Response:** The entire manuscript has undergone **thorough proofreading** to correct grammatical errors, improve sentence structure, and enhance clarity and flow. Redundant phrasing was removed, terminology was standardized (e.g., consistent use of “associated factors”), and long sentences were simplified in accordance with PLOS ONE style preferences. These revisions have been applied consistently across the abstract, main text, tables, and supplementary materials.

**REVIEWER COMMENTS**

**REVIEWER 1**

***Comment 1: Please define a school “term” and its typical duration.***

**Response:** We agree that defining a school “term” is essential for interpreting the measurement of chronic absenteeism. In Uganda, the academic year for the elementary (primary) and high schools is divided into three terms, each typically lasting approximately 12–13 weeks, excluding weekends and official school holidays. This corresponds to an average of about 89 instructional days per term. We have revised the manuscript to explicitly define a school term and its duration at the point where chronic absenteeism is operationalized.

Revised text: Methods: Study variables, Page: 6, Lines: 122-127

“The primary outcome was chronic school absenteeism, defined as missing ≥10% of expected school days in a term, consistent with UNICEF criteria (11). In Uganda, a school term typically lasts 12–13 weeks (~89 instructional days), excluding weekends and official school holidays (14). This percentage was calculated by dividing the number of days missed by the total number of expected school days. School days missed were recorded as an integer based on caregiver or participant self-report for the previous school term.”

***Comment 2:*** ***The need for or lack thereof for transportation to school can often be a barrier-was this a potential concern in this region or population? Any urban vs rural differences?***

Response: Transportation challenges are indeed an important determinant of school absenteeism, particularly in low-resource settings. In the present study, the majority of participants were drawn from an urban and peri-urban population, reflecting the catchment area of Mulago National Referral Hospital. Most children attended schools within reasonable walking distance or relied on informal, low-cost transport arrangements (e.g., walking, public motorcycles and minibuses(taxis)), which are common in this setting. While transportation difficulties were not systematically measured as a standalone variable in this study, we acknowledge that transportation barriers, especially in rural settings, may further exacerbate absenteeism among children with sickle cell disease. We have therefore clarified the urban/peri-urban context of the study population and explicitly acknowledged transportation as an unmeasured contextual factor that may limit generalizability to rural populations.

Revised text: Discussion-limitations, Page: 18-19, Lines: 354-358

“In addition, most participants were recruited from urban and peri-urban areas within the catchment of the national referral hospital. Transportation barriers were not directly assessed; however, such challenges may be more pronounced in rural settings and could further contribute to school absenteeism among children living with chronic illnesses such as SCD.”

***Comment 3: Please elaborate on availability of online/virtual schooling or tutoring services****.*

Response: In Uganda, access to online or virtual education remains very limited. During the study period, structured online schooling or remote tutoring was largely unavailable to most families attending the Sickle Cell Clinic due to constraints related to internet access, digital devices, electricity reliability, and cost. As a result, children who miss school because of illness or hospitalization generally lack formal mechanisms for remote learning or academic catch-up. This limited availability of virtual schooling likely amplifies the educational impact of illness-related absenteeism for children with sickle cell disease. We have expanded the Discussion to reflect this contextual reality and to emphasize the need for alternative learning support strategies in low-resource settings.

Revised text: Section: Discussion, Page: 18, Lines:329-332

“Access to online or virtual schooling remains limited in Uganda. During periods of illness or hospitalization, most children lack structured remote learning or tutoring options, which may intensify the educational consequences of school absenteeism among learners with SCD.”

**REVIEWER 2**

***Comment 1: In the abstract and the manuscript, the authors use the words "predictors" and "associated factors" interchangeably. However, this is not acceptable. Predictors are only measurable in a cohort design only as opposed to associated factors that can be measured cross-sectionally..***

Response: As this is a cross-sectional study, the use of the term “predictors” was inappropriate. We have therefore removed the term “predictors” throughout the manuscript and replaced it consistently with “associated factors”, including in the abstract, methods, results, discussion, tables, and conclusions.

Revised text: Sections affected include: Abstract, Page 2, lines: 26-27

“This study assessed the prevalence and associated factors of chronic absenteeism among children and adolescents with SCD to inform education and health policy.”

***Comment 2: In essence, this is a matched case control study and should be presented as such right from the title and the body. The SCD participants are the cases and the matched peers are the controls***

Response: We respectfully maintain that the study is appropriately classified as a comparative cross-sectional study and not a case-control study. We recognize that the inclusion of two groups may resemble a case-control structure; however, the recruitment process and analytical framework align with cross-sectional methodology.

Participants were recruited based on exposure status (presence or absence of sickle cell disease) rather than outcome status (chronic absenteeism). Children with SCD were consecutively enrolled from the Sickle Cell Clinic during routine visits. For each enrolled child with SCD, a sibling or peer without SCD of similar age was recruited as a comparison participant. Importantly, participants were not selected based on whether they had chronic absenteeism, and absenteeism status was unknown at the time of enrollment.

Both exposure (SCD status) and outcome (chronic absenteeism during the previous school term) were assessed concurrently at a single point in time. No retrospective identification of cases and controls based on absenteeism occurred, and no matching or conditional analytical techniques typical of case-control designs were applied.

Revised text: Section**:** Methods - *Study design and setting*, Page**:** 5, Lines: 93-102

“This was a comparative cross-sectional study conducted among children aged 6–19 years. Participants were recruited based on sickle cell disease (SCD) status rather than absenteeism status. Children with SCD were consecutively enrolled during routine clinic visits at the national referral hospital’s Sickle Cell Clinic. For each enrolled child with SCD, a sibling or age-comparable peer without SCD was recruited from the same household or community. Chronic absenteeism was assessed at the time of interview based on school attendance during the previous term. Participants were not selected based on absenteeism status, and no retrospective identification of cases and controls was performed.”

***Comment 3: In the sample size calculation, it is not clear what "q1" and "q2" stand for. Sample size for two proportion comparison study requires only "p1" (Expected proportion of chronic absenteeism in the SCD participants) and "p2" (Expected proportion of chronic absenteeism in the controls).***

Response: We agree that the notation used in the original manuscript was non-standard and potentially confusing. The sample size calculation was based on a comparison of two proportions, and the appropriate parameters are p₁ (expected proportion of chronic absenteeism among children with sickle cell disease) and p₂ (expected proportion among siblings/peers without sickle cell disease). We assumed 55% of children would have SCD and 45% normal siblings. Of the children with SCD, we assumed 38% would chronically miss school compared to 19% of their peers. We considered a 5% level of significance. We have revised the manuscript to remove the terms q₁ and q₂ entirely, replaced them with standard two-proportion notation (p₁ and p₂), and defined these parameters in the Methods section.

Revised text: Section: Methods*-Data analysis* Page: 8, Lines: 179-187

“With chronic school absenteeism as the primary outcome, the required sample size was calculated to detect a difference in the proportion of chronic absenteeism between children with sickle cell disease (SCD) and their siblings or peers without SCD. The calculation was based on a two-proportion comparison formula. We assumed an expected prevalence of chronic absenteeism of 38% among children with SCD (p₁ = 0.38) and 19% among siblings/peers (p₂ = 0.19), informed by previously published studies conducted in similar settings (12). A 5% level of significance (Zα = 1.96) and 80% power (Zβ = 0.84) were applied. The resulting minimum sample size was 179 participants per group.

***Comment 4. In the same sample size calculation, there is no reference or explanation for how the p1 and p2 were obtained since these were supposed to be predetermined.***

**Response:** We have now clarified that the assumed proportions were derived from **previous published studies** reporting absenteeism among children with SCD and their peers in similar settings. Appropriate references have been added to justify these assumptions.

**Revised text: Section:** Methods – Data analysis, **Page:** 8, **Lines:** 184-186

“The assumed proportions (p₁ and p₂) were informed by prior studies reporting school absenteeism among children with SCD and comparison groups in similar contexts (12)”

***Comment 5. In the results, table one should strictly contain baseline demographic characteristics and should not contain the "results". Eg number of school days missed, reasons for missing school etc***

Response: Table 1 has been revised to include baseline sociodemographic and school characteristics only. All outcome-related variables, including number of school days missed and reasons for absenteeism, have been removed from Table 1 and the outcomes left as text only.

Revised tables: Table 1: Baseline characteristics only, Pages: 10: Lines-225

| **Characteristic** | **With SCD (N=179),**  **n (%) or Median (IQR)** | **Sibling/peer (N=179),**  **n (%) or Median (IQR)** |
| --- | --- | --- |
| **Sex**  Male  Female | 90 (50.3)  89 (49.7) | 76 (42.5)  103 (57.5) |
| **Current age**  Median (IQR), years | 11(9-13) | 11(9-14) |
| **Same biological parents as child with SCD** | | |
| No  Yes |  | 38 (21.2)  141 (78.8) |
| **Highest level of education** |  |  |
| Nursery  Primary  Secondary  Tertiary | 19 (10.6)  133 (74.3)  27 (15.1)  0 (0.0) | 13 (7.3)  123 (68.7)  42 (23.5)  1 (0.5) |
| **Day or Boarding School** |  |  |
| Day  Boarding | 158 (88.3)  21 (11.7) | 127 (71.0)  52 (29.0) |
| **Private or public school** |  |  |
| Private | 137 (76.3) | 144 (80.5) |
| Public/Government | 42 (23.5) | 35 (19.5) |
| **Ever repeated class** |  |  |
| No | 135 (76.3) | 154 (86.0) |
| Yes | 42 (23.7) | 25 (14.0) |

***Comment 6. I see no use of table 2 in the results, which objective is this answering? We need results on the following***

***- Prevalence of chronic absenteeism in the two groups with their 95%CI and the comparison (prevalence ratio or OR with CI)***

***-Bi-variate and multivariate logistic regression on the associated factors (And this should be limited to only the SCD group since its entirely exploratory and the study is not powered for a comparison between the cases and controls).***

Response: We appreciate this guidance and have substantially reorganized the Results section. Specifically:

Table 2 is presented to show the clinical characteristics that influenced/associated with the primary outcome and the narrative has been described better in the title and the results.

Table 3 presents the prevalence of chronic absenteeism in the two groups with their 95%CI and the comparison (prevalence ratio or OR with CI)

Table 4 presents both the bi-variate and multivariate logistic regression on the associated factors limited to only the SCD

Revised text: Results. – Pages 13, lines: 251, 272, 229-229

“Characteristics of children with sickle cell disease and their association with school absenteeism during the previous term (n = 179)”

“Chronic school absenteeism was reported by 33% (95% CI: 26-40%, 59/179) of children with SCD compared to 6% (95% CI: 3-11%, 11/179) of their siblings/peers”

***Comment 7. Table 4 can be improved significantly, the crude and adjusted odds ratios can be presented concurrently in the same table (Again forget about the comparison as this is not useful), and on the rows, only present the positive (Yes) instead of one row for "Yes" and another row for "no' for the same variable eg only one row for "ever repeated a class?"***

Response: We would like to clarify that in Table 4, we want to show the reference categories and the percentage of children who missed school within each category. We have revised the table and removed the, No rows

Revised text: Table 4, page 15, lines: 272

| **Characteristic** |  | **Chronic absenteeism, Yes, n (%)** | **OR**  **(95% CI)** | ***P***  **value** | **aOR**  **(95% CI)** | ***p***  **value** |
| --- | --- | --- | --- | --- | --- | --- |
| **Gender** |  |  |  |  |  |  |
| Female |  | 24 (27.0) | 0.6 (0.3-1.1) | 0.091 | - | - |
| Male |  | 35 (38.9) | Reference |  |  |  |
| **School type** |  |  |  |  |  |  |
| Boarding |  | 4 (19.1) | 0.4 (0.1-1.4) | 0.158 | - | - |
| Day |  | 55 (34.8) | Reference |  |  |  |
| **Ever repeated class** | | | | | | |
| Yes |  | 20 (47.6) | 2.4 (1.2-4.9) | **0.016** | 2.5 (1.1-5.9) | **0.035** |
| **Household income sufficient to pay school fees on time** | | | | |  |  |
| Yes |  | 12 (25.5) | 0.6 (0.2-1.3) | 0.209 | - | - |
| **Children missing school due to the failure to pay fees** | | | | |  |  |
| Yes |  | 47 (36.4) | 1.8 (0.9-3.8) | 0.115 | - | - |
| **Mother's highest level of education** | | | |  |  |  |
| ≥Secondary |  | 31 (27.4) | 0.6 (0.3-1.1) | 0.120 | - | - |
| ≤Primary |  | 22 (39.3) | Reference |  |  |  |
| **Number of visits to the SCC** | | | |  |  |  |
| Mean (±SD) |  | 1.9 (±1.5) | 1.7 (1.3-2.2) | **<0.001** | 1.3 (1.0-1.8) | 0.076 |
| **Total number of times the child was admitted** | | | |  |  |  |
| Mean (±SD) |  | 0.9 (±0.8) | 3.9 (2.2-6.9) | **<0.001** | 3.6 (1.9-6.8) | **<0.001** |
| **Total number of episodes of pain crises** | | | |  |  |  |
| Mean (±SD) |  | 2.2 (±2.3) | 1.3 (1.1-1.5) | **0.005** | - | - |
| **Number of previous blood transfusions** | | | | | | |
| Mean (±SD) |  | 0.4 (±0.8) | 2.6 (1.3-5.0) | **0.005** | - | - |

***Comment 8:*** ***Limit the discussion and conclusion to what the study is powered for.***

Response: The Discussion and Conclusions have been tightened to focus strictly on findings supported by the study design and statistical power. Over-interpretation has been avoided, causal language has been removed, and conclusions are framed in terms of associations and implications rather than effects.

***Comment 9. Limitations: Please report the traditional limitations for case control study, and in your study, the cases were more likely to remember or have records as regards chronic absenteeism***

Response: Thank you very much for this comment. As clarified in our earlier response regarding study design, participants were recruited based on sickle cell disease (SCD) status rather than absenteeism status, and exposure and outcome were assessed concurrently. Nevertheless, we agree that several limitations traditionally discussed in case-control studies are relevant and should be explicitly acknowledged.

In particular, we recognize the potential for recall bias in the reporting of school absenteeism. Caregivers of children with SCD may be more likely to recall or maintain records of illness-related absences compared to caregivers of siblings or peers without SCD, introducing the possibility of differential recall. We have therefore expanded the Limitations subsection to address recall bias, potential differential misclassification, lack of temporality, residual confounding, and selection bias due to the inclusion of only children currently enrolled in school.

Revised text: Section**:** Discussion. Page**:** 18-19, Lines**:**344-362

“Nonetheless, limitations exist. First, the cross-sectional design limits the ability to establish temporal relationships between associated factors and chronic absenteeism; therefore, causal inferences cannot be made. Second, school absenteeism was assessed using caregiver or participant self-report for the previous school term, which may be subject to recall bias and misclassification. Caregivers of children with sickle cell disease may recall illness-related absences more readily than caregivers of comparison participants, potentially resulting in differential recall. Third, as with many observational studies, residual confounding from unmeasured variables cannot be excluded despite multivariable adjustment. Factors such as school-level support systems, teacher attitudes, and household socioeconomic dynamics that were not fully captured in this study may influence patterns of absenteeism. In addition, most participants were recruited from urban and peri-urban areas within the catchment of the national referral hospital. Transportation barriers were not directly assessed; however, such challenges may be more pronounced in rural settings and could further contribute to school absenteeism among children living with chronic illnesses such as SCD. Finally, participants were recruited from a clinical setting and included only children currently enrolled in school. Children with sickle cell disease who had permanently withdrawn from school were not captured, which may have led to underestimation of the true educational burden associated with the condition.”

We hope that we have satisfactorily addressed the reviewer’s comments and that the manuscript is now suitable for publication. We are grateful to the reviewers for their suggestions and believe these revisions have improved the clarity, interpretability, and readability of our manuscript.

Sincerely,

Nattimba Juliane Peninah

Corresponding author
