# Supplementary material for: School absenteeism among children and adolescents aged 6–19 years with sickle cell disease in Uganda: A comparative cross-sectional study
Source: PLoS One. 2026 May 29;21(5):e0350308. doi: 10.1371/journal.pone.0350308 (PMC13221043; doi:10.1371/journal.pone.0350308)
Supplement: Attachment — Submitted filename: Response_to_Reviewers_auresp_2.docx [file pone.0350308.s004.docx]

**MAKERERE** **UNIVERSITY**

**COLLEGE OF HEALTH SCIENCES**

**SCHOOL OF MEDICINE**

**DEPARTMENT OF PEDIATRICS AND CHILD HEALTH**

23^rd^ April 2026

Dr. Aloysius Gonzaga Mubuuke
Academic Editor
PLOS One

Dear Dr. Mubuuke,

On behalf of my co-authors, I am pleased to submit our responses to the reviewers’ comments for the manuscript titled **“School absenteeism among children and adolescents aged 6–19 years with sickle cell disease in Uganda: a comparative cross-sectional study”** (Manuscript ID: **PONE-D-25-63605**). We sincerely thank the Academic Editor and reviewers for their careful evaluation of our manuscript and for their valuable and constructive comments. We have carefully addressed each comment in detail below and revised the manuscript accordingly.

**EDITOR COMMENTS**

**Comment 1:** The paper has significantly improved and is benefit to the readership of PLOS ONE. The discussion has been strengthened. However, before the authors explain the strengths and limitations of the study towards the end of the discussion, they should include some key implications of the findings from this study first to clinicians who take care of these children with SCD and second, to the school administration authorities especially that they deal with these children while in school.

Response: We have revised the Discussion section to include a new paragraph on the practical implications of our findings before the strengths and limitations section. Specifically, we added implications for clinicians caring for children and adolescents with sickle cell disease (SCD), emphasizing routine screening for school absenteeism, early management of pain crises and hospitalizations, caregiver counseling, and psychosocial support. We also included implications for school administrators and education authorities, highlighting the need for flexible attendance policies, teacher sensitization, improved communication between schools and families, and academic support for learners with SCD.

Revised text: Discussion section, page 18-19, lines 341-360

“These findings have important implications for clinicians caring for children and adolescents with sickle cell disease. Routine clinical care should include screening for school absenteeism as part of holistic patient assessment, since frequent absence may signal worsening disease severity, psychosocial distress, or barriers to treatment adherence. Early identification and management of pain crises, infections, and other complications that lead to hospitalization may help reduce school disruption. Clinicians should also counsel caregivers on the importance of sustained school attendance and work with families to schedule routine clinic visits, where possible, outside critical school periods. Strengthening psychosocial support and referral systems for learners with repeated absenteeism may further improve both health and educational outcomes.

The findings also have important implications for school administrators and education authorities. School administrators should recognize sickle cell disease as a chronic health condition requiring flexible and supportive attendance policies rather than punitive responses to repeated absence. Improved communication between schools, caregivers, and healthcare providers may help schools better understand illness-related absenteeism and support timely academic catch-up. Teacher sensitization on the challenges faced by learners with SCD is important to reduce stigma, improve classroom support, and encourage retention in school. Where feasible, schools should strengthen school health programs and provide reasonable accommodations such as flexible assessment schedules, remedial learning support, and referral pathways for children experiencing frequent illness-related interruptions in learning.”

We hope we have satisfactorily addressed all comments from the Academic Editor and reviewers, and that the revised manuscript is now suitable for publication in PLOS ONE.

We are sincerely grateful for the valuable suggestions, which have helped improve the clarity, interpretability, and overall quality of our manuscript.

Sincerely,

Nattimba Juliane Peninah

Corresponding author
